# Multimodal ultrasound features for distinguishing classic and aggressive subtypes of papillary thyroid carcinoma

**DOI:** 10.3389/fendo.2025.1674109

**Published:** 2025-10-01

**Authors:** Bixue Deng, Jing Zhong, Yu Zhuang, Jiamin Chen, Jiayi Hong, Xiaofeng Qin, Zhongzhen Su, Jiahui Zhang, Fei Chen, Xin Wen

**Affiliations:** Department of Ultrasound, The Fifth Affiliated Hospital, Sun Yat-sen University, Zhuhai, China

**Keywords:** thyroid, cancer, papillary, carcinoma, multimodal ultrasonography, classic subtype, aggressive subtypes

## Abstract

**Background:**

To compare the sonographic features of papillary thyroid carcinoma (PTC) between classic and aggressive PTC subtypes to determine whether multimodal ultrasound (US) can aid in differentiating particular subtypes.

**Methods:**

The retrospective cohort study included patients with histologically proven PTCs according to the World Health Organization classification of thyroid neoplasms between December 2022 and October 2024. Imaging findings were evaluated using the International Expert Consensus on US Lexicon for Thyroid Nodules. Associations between US features and intrinsic subtypes were assessed by the χ^2^ or Fisher’s exact test.

**Results:**

Overall, 295 patients with 320 nodules (74 males with 81 nodules and 221 females with 239 nodules) were included. There were 279 classic PTC (87.2%), 26 tall cell (8.1%), 11 hobnail (3.4%), one columnar cell (0.3%), one solid (0.3%), and two diffuse sclerosing subtypes (0.6%). Regarding US features, direction of growth, extrathyroidal extension (ETE), calcifications, and color Doppler flow imaging significantly differed among the PTC subtypes. Tall cell subtype PTCs exhibited the highest prevalence of taller-than-wide shapes (*p*<0.001) and the absence of echogenic foci (*p*=0.047). ETE was not observed in hobnail subtype PTCs (*p* =0.008). The vascularity of classic and tall cell subtype PTCs usually presented as absent or rim blood signals, while the hobnail subtype commonly had vessels inside the nodule (*p*=0.017). All subtypes of PTC demonstrated similarly high stiffness values on SWE. The mean Emax, Emean, and Emin were 64.6 ± 38.2 kPa, 45.1 ± 29.6 kPa, and 27.7 ± 20.7 kPa, respectively.

**Conclusion:**

There were significant differences and several trends in the US characteristics of different intrinsic subtypes, providing imaging diagnostic criteria to assist in managing individuals with PTC.

## 1 Introduction

Papillary thyroid carcinoma (PTC), the most common follicular cell-driven neoplasm, constitutes 89.1% of thyroid malignancies (1). Although classic PTC (C-PTC) is acknowledged as the paradigm for all PTC subtypes, several other subtypes have been characterized based on their cell types, molecular profiles, and aggressiveness. The 2022 World Health Organization (WHO) classification identified several aggressive PTC variants, including tall cell (TC-), columnar cell (CC-), hobnail (HN-), diffuse sclerosing (DS-), and solid subtypes (S-), which are associated with poorer outcomes and may require different management approaches (2). Cytologically suspicious aggressive subtypes warrant immediate surgery, whereas active surveillance (AS) is adopted for low-risk PTC (3). However, the diagnostic performance of fine-needle aspiration (FNA) is highly dependent on sample adequacy, the operator’s technical skill, and the cytopathologist’s expertise (4), with approximately 20%-25% of FNA results fall into indeterminate categories (5). Therefore, identifying diagnostic features for aggressive subtypes may be critical in deciding the clinical management of thyroid nodules.

Ultrasonography (US) is pivotal in thyroid nodule evaluation, providing real-time, non-invasive assessment of morphological features and characterization. Recent technological advancements in color Doppler flow imaging (CDFI) and shear wave elastography (SWE) enhance diagnostic capabilities for risk stratification (6, 7). CDFI provides valuable information on vascular patterns and angiogenesis, while SWE offers a quantitative assessment of tissue elasticity (8, 9), which may serve as potential biomarkers for differentiating PTC subtypes. The combination of multiple ultrasound techniques can improve diagnostic performance and mitigate the limitations inherent to each modality. Multimodal ultrasound, integrating grayscale ultrasound (GUS), elastography, and color Doppler/contrast-enhanced ultrasound (CDFI/CEUS), enables multidimensional evaluation of thyroid nodules by combining morphological characteristics, tissue stiffness, and vascular patterns. Several studies have demonstrated that this comprehensive approach achieves superior diagnostic accuracy compared with any single or dual modality, further guiding clinical decision-making for thyroid nodules and helping to avoid unnecessary FNA procedures (10, 11). Given the different clinical behaviors and prognosis of PTC subtypes, recognizing the multimodal US features of PTC subtypes has become increasingly important in current diagnostic practices.

Nevertheless, knowledge of the differences in US characteristics between classic PTC and aggressive PTC subtypes is limited. The current literature predominantly focuses on greyscale US features in PTC subtypes, which have been compared to C-PTC (13–16). To our knowledge, no article has systematically compared multimodal US features between C-PTC and aggressive histologically-proven PTC subtypes based on the 2022 WHO classification of thyroid neoplasms. Therefore, this study aimed to analyze and compare multimodal US features across PTC subtypes considering the novel classification to determine whether US can aid in the differentiation of particular subtypes.

## 2 Methods

The study was performed in agreement with the Declaration of Helsinki (2003-036B). The Institutional Review Boards at the Fifth Affiliated Hospital of Sun Yat-sen University approved the study, with waivers for written informed consent from the patients (No. 36K-1 in 2023).

### 2.1 Patient selection

The retrospective cohort study included consecutive patients who underwent preoperative thyroid gland US examination followed by thyroid surgery between December 2022 and October 2024. The inclusion criteria were as follows: (a) histopathological confirmation of C-PTC or aggressive PTC subtypes; (b) comprehensive US examination reports with multimodal imaging data; (c) demographic and clinicopathologic data records. The exclusion criteria were as follows: (a) thyroid nodules with a maximum diameter <5mm; (b) poor image quality due to motion artifacts (e.g., coughing, speech-related movements), intermittent scanning and improper probe compression; and (c) nodules with coarse calcifications obscured by shadowing, and (d) ambiguous image-to-pathology correlation. Clinicopathological characteristics including patient age, sex, surgical method, lymphatic metastasis and maximum size of nodules, were recorded.

### 2.2 Examination procedures

Thyroid nodules were examined using a Supersonic Aixplorer system (SuperSonic Imagine, Aix-en-Provence, France) equipped with a 5-14 MHz linear transducer. Two experienced radiologists (B.X.D., 5 years; X.W., 15 years) independently evaluated multimodal ultrasound images while blinded to clinical and pathological details. Discrepancies were resolved through review by a senior radiologist (S.Z.Z., 25 years), whose consensus decision was final. Postoperative pathology was regarded as the gold standard. For patients with multiple thyroid nodules, lesions were concordantly identified by both US and pathology experts.

### 2.3 Evaluation criteria

GUS features were recorded according to the International Expert Consensus on US Lexicon for Thyroid Nodules (17), including composition, echogenicity, margin, direction of growth, calcifications, and extrathyroidal extension. The detailed radiological definitions of each US feature are provided in **Supplementary Table S1**. Each thyroid nodule was classified into the China Thyroid Imaging Reporting and Data System (C-TIRADS) categories 3-5 (18). C-TIRADS assigned levels of malignancy risk to different patterns, totaling five features: solid composition, microcalcifications, markedly hypoechoic, ill-defined/irregular margins or extrathyroidal extensions, and vertical orientation. Each of these features scored +1 point. Comet-tail artifacts were considered a sign of a benign nodule and scored –1 point. Every category and malignant rate was based on the points in C-TIRADS (**Supplementary Table S2**).

Next, color Doppler analysis was performed. The vascularity of the nodules was categorized according to the CHAMMAS classification system: I, no blood flow signal; II, exclusively perinodular vascularity; III, perinodular blood flow ≥ central blood flow; IV, marked central blood flow and less marked perinodular blood flow; and V, exclusively central vascularity (19).

Finally, SWE measurements were performed using the device manufacturer’s “Q-Box” quantification tool to position a 3mm diameter region of interest (ROI). We measured the mean (Emean), minimum (Emin), maximum (Emax), and standard deviation (Esd) of the elasticity index (kPa). The nodule ROI was positioned at the hardest part of the nodule, avoiding calcifications, cystic components, and areas with color defects, while the thyroid ROI was placed in the surrounding thyroid parenchyma at the same depth to calculate the elasticity ratio (ER). This enables a comparison between the mean elasticity of the nodule and the parenchyma. Three SWE images were obtained for each target nodule.

### 2.4 Histological analysis

All 320 histological specimens of PTC thyroid nodules from 295 patients had a conclusive pathological diagnosis confirmed by senior pathologists based on the 2022 WHO classification, with additional consideration of published evidence (20). C-PTC was defined by the presence of well-formed papillae lined with tumor cells that showed characteristic nuclear features, including enlargement, grooving, and peripheral margination of chromatin. A diagnosis of TC-PTC requires that at least 30% of tumor cells exhibit a height three times their width and possess an eosinophilic cytoplasm, along with easily identifiable nuclear features of PTC, even when mixed with classic or follicular growth patterns. A tumor was classified as the HN-PTC when more than 30% of tumor cells were hobnail cells with enlarged nuclei, bulging from the apical surface. The S-PTC was identified by a solid, trabecular, or nested growth pattern with intervening fibrovascular bands, rarely showing dense sclerosis and tumor necrosis. A tumor composed of diffuse unilateral or bilateral involvement with fibrous stroma, dense lymphocytic infiltration, and abundant psammoma bodies was defined as DS-PTC. The CC-PTC was characterized by a papillary growth pattern admixed with follicles, lined by columnar cells with pale to eosinophilic cytoplasm, and showing subnuclear vacuoles. In cases where more than one PTC subtype was present within the same nodule, the lesion was classified according to the more aggressive component.

Histopathological reports of all nodules were evaluated to document the tumor size, tumor focality, location, capsular presence, extrathyroidal extension status, vascular/lymphatic invasion, lymph node metastasis, surgical margin, and thyroid parenchymal features.

### 2.5 Statistical analysis

Categorical variables are presented as counts and percentages, and continuous variables as the mean and standard deviation (SD) or median and interquartile range (IQR) based on the normality assumption assessed by the Shapiro-Wilk test. One-way ANOVA and Kruskal-Wallis tests evaluated age and tumor size correlations with the intrinsic subtypes. The associations between US features and intrinsic subtypes were assessed by χ2 tests or Fisher’s exact test and Bonferroni corrections. Due to limited cases, the CC-, S-, and DS- subtypes were excluded from the statistical comparison of age, size, and ultrasonographic features. Cramer’s V was calculated for the effective size (21), indicating the strength of the association, which was categorized as follows: weak (0.1-0.2), moderate (0.2-0.4), or strong (0.4 or greater) (22). The Cohen’s Kappa was used for interobserver agreement of ultrasound features. The Kappa-value mirrors the interobserver agreement, with values 0.01-0.20 slight agreement, 0.21-0.40 fair agreement, 0.41-0.60 moderate agreement, 0.61-0.80 substantial agreement and 0.81-0.99 almost perfect agreement (23).

All analyses were performed with R version 4.4.0 (R Foundation for Statistical Computing, Vienna, Austria), with a *p-value*<0.05 as the threshold for statistical significance.

## 3 Results

### 3.1 Patients and US characteristics

Between December 2022 and October 2024, 387 nodules from 350 patients were confirmed as pathologically PTCs after surgical resection. A total of 67 nodules were excluded due to suboptimal or incomplete imaging quality (21 patients with 23 nodules), discordance between ultrasonographic and pathological findings regarding lesion location or size (8 patients with 18 nodules), nodules with coarse calcifications obscured by shadowing (5 patients with 5 nodules), and maximum diameter <5mm (26 patients with 26 nodules) (**Supplementary Figure S1**).

Overall, 295 patients, including 74 (25.1%) males with 81 (25.3%) nodules and 221 (74.9%) females with 239 (74.7%) nodules, were included. The histopathological diagnoses of the resected PTC specimens were as follows: C-PTC (279/320, 87.2%), TC-PTC (26/320, 8.1%), HN-PTC (11/320, 3.4%), CC-PTC (1/320, 0.3%), SS-PTC (1/320, 0.3%), DS-PTC (2/320, 0.6%). Both DS-PTC cases in our cohort presented as nodular lesions on ultrasonography. The demographic characteristics of the study population are presented in **Table 1**. The age distribution demonstrated a peak incidence in the 30–39 years age group. There was a significant difference between the mean ages of patients with different PTC subtypes (*p*=0.004), the highest mean age was detected in the patients with TC-PTC, while the lowest age was observed in a 32-year-old patient with S-PTC, who was excluded from statistical comparison of mean ages since there was only one case. The US findings of the various PTC subtypes are summarized in **Table 2**, according to the International Expert Consensus on US Lexicon. The maximum nodular diameter measured using sonography differed significantly, with C-PTC lesions being smaller than those of TC-PTC and HN-PTC subtype lesions (*p*=003). Composition, echogenicity, and margin features did not reach a significant difference among subtypes. Most nodules presented as solid and mildly hypoechoic, in line with typical malignant characteristics. With regard to margins, the majority were smooth or irregular, whereas ill-defined margins were relatively uncommon. Significant differences were detected in the direction of growth, calcification pattern, extrathyroidal extension, and CDFI features. Significant differences were detected in the direction of growth, calcifications pattern, extrathyroidal extension, and CDFI features. Regarding the vascularity on US imaging, nodules were characterized by peripheral or absent, while intra-nodular vascularity was relatively uncommon. All subtypes of PTC demonstrated similarly high stiffness values on SWE. The mean Emax, Emean, and Emin were 64.6 ± 38.2 kPa, 45.1 ± 29.6 kPa, and 27.7 ± 20.7 kPa, respectively, while the mean Esd and ER were 8.6 ± 6.2 kPa and 2.2 ± 3.2. A detailed summary of multimodal ultrasonographic features is provided in **Supplementary Table S3**.

**Table 1:** Demographic characteristics of the study population.

Variable	Overall	Classic PTC	Tall cell subtype	Hobnail subtype	Column subtype	Solid subtype	Diffuse sclerosing subtype	*p* value
Number of patients	295	255	25	11	1	1	2	
Age (year)	40.2 ± 10.9	39.9 ± 10.8	45.73 ± 11.45	34.36 ± 8.95	51	32	31.5 ± 6.5	**0.004** ^a^
<30	47 (15.9%)	41 (16.1%)	1 (4.0%)	4 (36.4%)	0	0	1 (50%)	
30-39	111 (37.6%)	96 (37.6%)	7 (28.0%)	6 (54.5%)	0	1 (100%)	1 (50%)	
40-49	69 (23.4%)	65 (25.5%)	4 (16.0%)	0	0	0	0	
50-59	51 (17.3%)	39 (15.3%)	10 (40.0%)	1 (9.1%)	1 (100%)	0	0	
≥60	17 (5.8%)	14 (5.5%)	3 (12.0%)	0	0	0	0	
Sex								0.248
Male	74 (25.1%)	61 (23.9%)	7 (28.0%)	6 (54.6%)	0	0	0	
Female	221 (74.9%)	194 (76.1%)	18 (72.0%)	5 (45.4%)	1 (100%)	1 (100%)	2 (100%)	
Lymphatic metastasis								0.138
Absent	130 (44.1%)	116 (45.5%)	10 (40.0%)	2 (18.2%)	1 (100%)	1 (100%)	0	
Present	165 (55.9%)	139 (54.5%)	15 (60.0%)	9 (81.8%)	0	0	2 (100%)	
Surgery								0.068
Thyroid lobectomy	186 (63.1%)	165 (64.7%)	11 (44.0%)	8 (72.7%)	1 (100%)	1 (100%)	0	
Total thyroidectomy	109 (36.9%)	90 (35.3%)	14 (56.0%)	3 (27.3%)	0	0	2 (100%)	
Number of nodules	320	279	26	11	1	1	2	
C-TIRADS								0.135^a^
3	4 (1.3%)	3 (1.1%)	0 (0%)	1 (9.1%)	0	0	0	
4A	21 (6.6%)	17 (6.1%)	1 (3.8%)	2 (18.2%)	1 (100%)	0	0	
4B	96 (30.0%)	85 (30.5%)	9 (34.6%)	2 (18.2%)	0	0	0	
4C	180 (56.3%)	159 (57.0%)	15 (57.7%)	4 (36.4%)	0	1 (100%)	1 (50.0%)	
5	19 (5.9%)	15 (5.4%)	1 (3.8%)	2 (18.2%)	0	0	1 (50.0%)	

Values are presented as mean ± SD, or count (%).

PTC, papillary thyroid carcinoma.

a The subtypes including column subtype, solid subtype and diffuse sclerosing subtype were excluded from the age and C-TIRADS comparison analysis owing to the small sample size.

Bold values indicate statistical significance (p < 0.05).

**Table 2:** Comparison of the sonographic features of the nodules having different histopathologic diagnoses.

Subtype US features	Overall (n=316)	Classic PTC (n=279)	Tall cell subtype (n=26)	Hobnail subtype (n=11)	*p* value
Diameter	11.6 ± 7.5	11.2 ± 7.1	11.8 ± 5.3	21.5 ± 14.4	**0.003**
Composition					0.165
Solid	312 (98.7%)	276 (98.9%)	26 (100%)	10 (90.9%)	
Mixed solid and cystic	4 (1.3%)	3 (1.1%)	0 (0%)	1 (9.1%)	
Echogenicity					0.146
Hyperechoic or isoechoic	12 (3.8%)	11 (3.9%)	0 (0%)	1 (9.1%)	
Mildly hypoechoic	253 (80.1%)	227 (81.4%)	18 (69.2%)	8 (72.7%)	
Markedly hypoechoic	51 (16.1%)	41 (14.7%)	8 (30.8%)	2 (18.2%)	
Margin					0.765
Smooth	129 (40.8%)	117 (41.9%)	8 (30.8%)	4 (36.4%)	
Ill-defined	54 (17.1%)	48 (17.2%)	4 (15.4%)	2 (18.2%)	
Irregular	133 (42.1%)	114 (40.9%)	14 (53.8%)	5 (45.4%)	
Direction of growth					**<0.001**
Wider-than-tall	142 (44.9%)	130 (46.6%)	4 (15.4%)	8 (72.7%)	
Taller-than-wide	174 (55.1%)	149 (53.4%)	22 (84.6%)	3 (27.3%)	
Calcifications					**0.047**
None	173 (54.8%)	151 (54.1%)	19 (73.1%)	3 (27.3%)	
Macrocalcifications	6 (1.9%)	5 (1.8%)	0 (0%)	1 (9.1%)	
Microcalcifications	123 (38.9%)	111 (39.8%)	5 (19.2%)	7 (63.6%)	
Mixed calcifications	14 (4.4%)	12 (4.3%)	2 (7.7%)	0 (0%)	
Extrathyroidal extension					**0.006**
Absent	235 (74.4%)	210 (75.3%)	14 (53.8%)	11 (100%)	
Present	81 (25.6%)	69 (24.7%)	12 (46.2%)	0 (0%)	
CDFI					**0.015**
I	125 (49.6%)	114 (40.9%)	9 (34.6%)	2 (18.2%)	
II	97 (30.7%)	87 (31.2%)	10 (38.5%)	0 (0%)	
III	58 (18.4%)	47 (16.9%)	6 (23.1%)	5 (45.5%)	
IV	34 (10.8%)	29 (10.4%)	1 (3.8%)	4 (36.4%)	
V	2 (0.6%)	2 (0.7%)	0 (0%)	0 (0%)	
SWE					
Emax (kPa)	64.6 ± 38.2	63.3 ± 37.3	69.6 ± 32.6	86.3 ± 60.5	0.310
Emean (kPa)	45.1 ± 29.6	44.1 ± 28.8	49.5 ± 26.5	60.1 ± 45.3	0.256
Emin (kPa)	27.7 ± 20.7	27.3 ± 20.8	31.0 ± 21.2	30.7 ± 16.6	0.294
Esd (kPa)	8.6 ± 6.2	8.4 ± 6.0	9.1 ± 5.3	11.4 ± 12.5	0.739
ER	2.2 ± 3.2	2.2 ± 3.4	2.0 ± 0.9	2.3 ± 1.1	0.465

Values are presented as mean ± SD or count (%).

PTC, papillary thyroid carcinoma; CDFI, color Doppler flow imaging; SWE, Shear wave elastography.

CDFI: I, absence of signal blood flow; II, exclusively perinodular blood flow; III, perinodular blood flow equal to or greater than intranodular blood flow; IV, marked intranodular blood flow and less significant perinodular blood flow; V, exclusively intranodular blood flow.

Bold values indicate statistical significance (p < 0.05).

### 3.2 Correlation of US features between classic PTCs and aggressive PTC intrinsic subtypes

**Figure 1:**
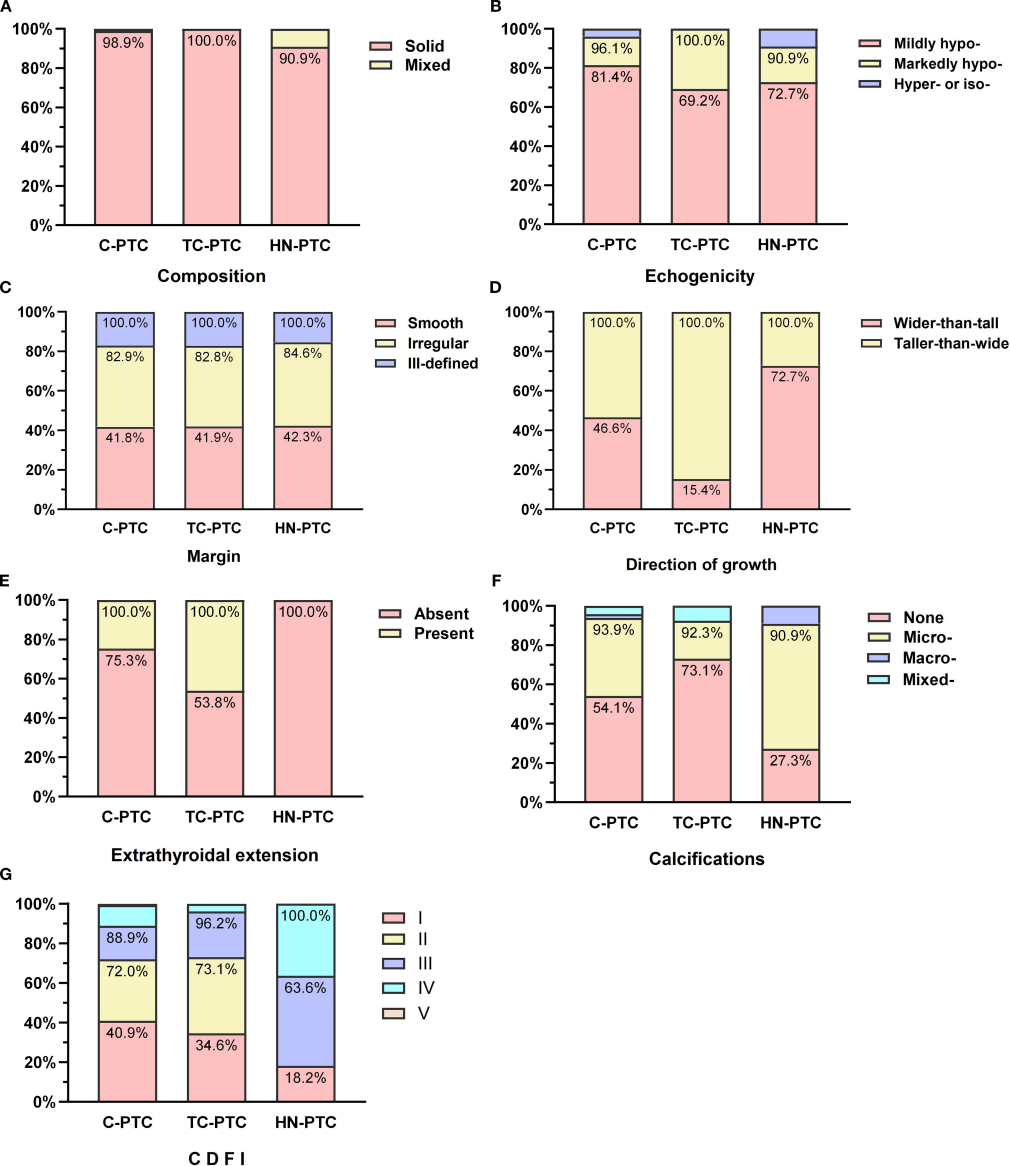
Comparisons of ultrasonographic features in classic PTC and aggressive subtypes: Composition **(A)**, Echogenicity **(B)**, Margin **(C)**, Direction of growth **(D)**, Calcifications **(E)**, Extrathyroidal extension **(F)**, Color Doppler flow imaging (CDFI) **(G)**.

**Figure 2:**
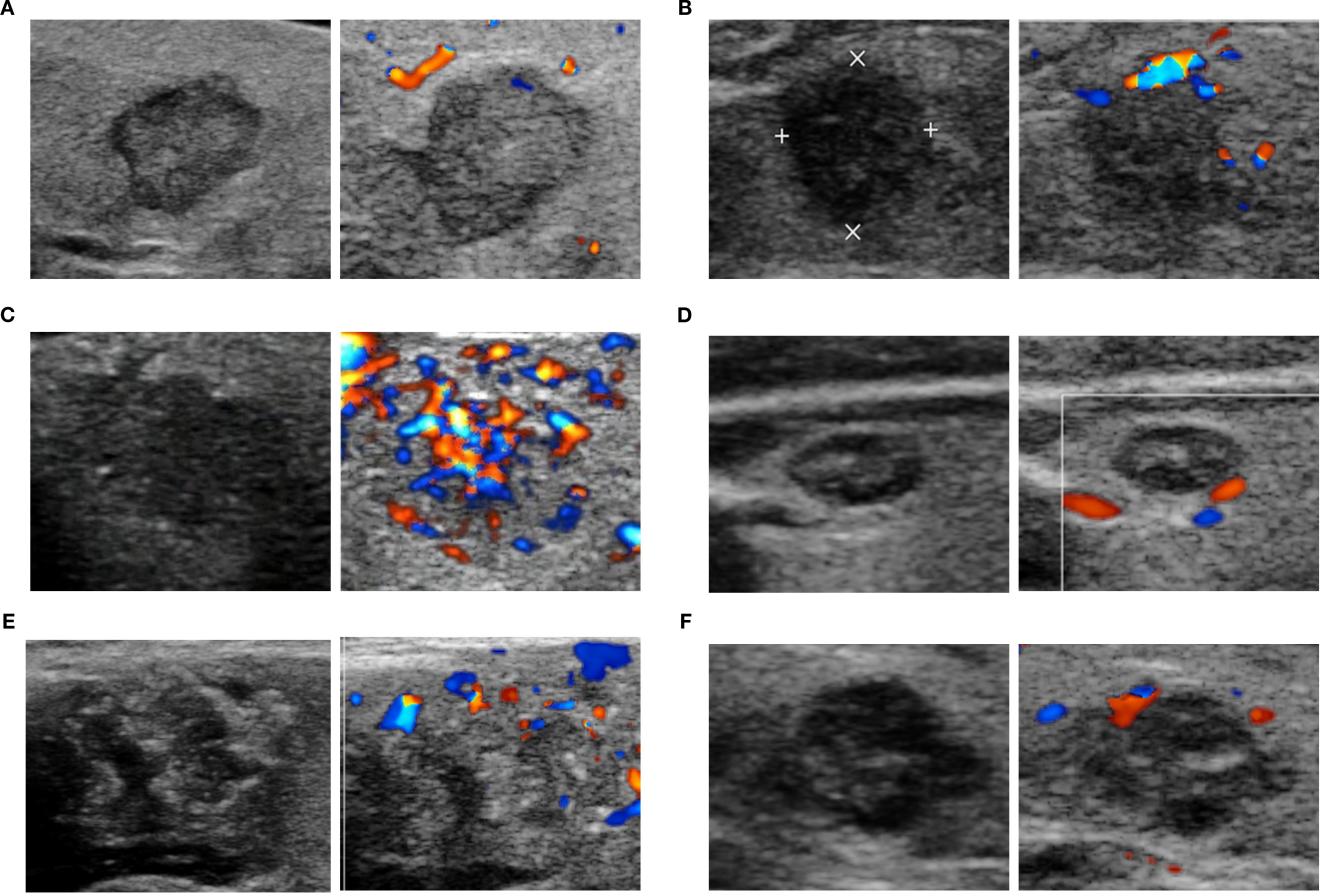
The representative ultrasonographic appearance of classic PTC and aggressive PTC. C-PTC **(A)** a hypoechoic nodule with solid composition, smooth margin, taller-than-wide shape, and absent of calcifications and blood flow signal (Chammas I); TC-PTC **(B)** a markedly hypoechoic nodule with solid composition, irregular margin, taller-than-wide shape, absence of calcifications and peripheral vascularity (Chammas II); HN-PTC **(C)** a hypoechoic nodule with solid composition, irregular margin, wider-than-tall shape, microcalcifications and internal blood signals (Chammas IV); CC-PTC **(D)** a hypoechoic and complex nodule with solid composition, smooth margin, wider-than-tall shape, microcalcifications and absence of signal blood flow (Chammas I); DS-PTC **(E)** a hypoechoic and complex nodule with solid composition, irregular margin, wider-than-tall shape, microcalcifications and peripheral vascularity (Chammas II); Solid PTC **(F)** a hypoechoic nodule with solid composition, irregular margin, wider-than-tall shape, microcalcifications and peripheral vascularity (Chammas II).

In total, 320 nodules met the eligibility criteria (US and relevant histopathological features available), and 316 nodules were included in the correlation analysis. The feature distributions of each subtype are presented in **Figure 1**, and the further pairwise comparisons of PTC subtypes are presented in **Supplementary Tables S4**-**S7**. The direction of growth differed significantly between TC-PTCs and both C-PTCs and HN-PTCs (*p*=0.005, *p*=0.004). HN-PTCs were characterized by calcifications, especially microcalcifications. Compared to the HN-PTCs group, calcifications were relatively uncommon in TC-PTCs (*p*=0.027). Although statistical comparison was not feasible due to the small sample size, calcifications were absent in the only cases of CC-PTC and S-PTC, whereas both DS-PTCs exhibited microcalcifications. C-PTCs and TC-PTCs presented a greater tendency for extrathyroidal extension, while all HN-PTCs had capsule-distant margins (*p*=0.006). Additionally, a significant difference was observed between TC-PTCs and HN-PTCs (*p*=0.020). The vascularity of C-PTCs and TC-PTCs usually presented as absent or rim blood signals, while HN-PTCs commonly had vessels inside the nodule (*p*=0.007, *p*=0.014). The vascular pattern of CC-PTCs, DS-PTCs, and S-PTCs was similar to C-PTCs. Despite not reaching statistical significance, higher elasticity parameters were an important index for PTC. The typical US appearance of each subtype is shown in **Figure 2**.

To further evaluate the strength of these associations, effect sizes were calculated using Cramer’s V. The direction of growth showed the strongest association with PTC subtypes (V=0.20, 95% CI: 0.08-0.31, *p*=0.002), while extrathyroidal extension (V=0.17, 95% CI: 0.05-0.28, *p*=0.008) and CDFI (V=0.17, 95% CI: 0.03-0.22, *p*=0.017) exhibited weak but statistically significant associations (**Supplementary Table S8**).

### 3.3 Interobserver agreement of US features

All Kappa-values were greater than 0.40 (all p < 0.001). The level of agreement among US features was substantial for calcifications (Kappa = 0.63, 95% Cl: 0.48-0.79) of nodules and for the orientation was high (Kappa = 0.85, 95% Cl: 0.71-0.97) (**Supplementary Table S9**).

## 4 Discussion

Given that a clinically feasible AS strategy has been increasingly adopted for PTC, recognizing PTC subtypes has become pivotal, as patients with high-risk PTCs are unsuitable for AS (3, 12). With improved imaging technology and resolution, US has become a reliable modality for differentiating the intrinsic subtypes of thyroid carcinoma (6, 24, 25). We present the multimodal US findings between C-PTC and aggressive PTC subtypes that can aid in differentiating subtypes with unfavorable outcomes from those with indolent clinical behavior.

The age distribution of patients with PTC included in our cohort aligns with previous studies (13, 16, 26), regardless of variations in race and publication years. Most PTCs were classified under C-TIRADS category 4C, suggesting a malignancy risk of 50%-90%. Consistent with this finding, Baek et al. also reported that the majority of PTCs were classified under K-TIRADS category 5 with malignancy risk > 60% (14). In our cohort, the prevalence of lymph node metastasis in aggressive PTC subtypes was higher than in classic PTC, although the difference was not statistically significant. This trend was generally consistent with previous studies (27). Simultaneously, total thyroidectomy was performed more often in aggressive PTC subtypes. “Given the uncertain prognostic value of histological variants as independent factors, clinical decision-making regarding surgical extent and adjuvant therapy should prioritize established prognosticators, including tumor dimensions and US characteristics.

In our cohort, the TC-PTC variant represented the predominant subtype among aggressive PTCs; however, it was previously reported to be an uncommon subtype, accounting for 1–19% of PTCs (28). Nine nodules contained both tall cell and either classic or follicular subtypes. Consistent with prior studies, we classified such lesions as TC-PTC when the tall cell component was present, given its recognized prognostic significance (29). In accordance with previous studies, TC-PTCs exhibited a solid composition, hypoechoic or markedly hypoechoic pattern, a taller-than-wide shape, absence of calcifications, and peripheral vascularity (16, 30). We observed significant differences in orientation, calcifications, and extrathyroidal extension between TC-PTCs and C-PTCs. Similarly, Sgrò et al. reported that calcifications were more common in C-PTC than in TC-PTC (30). These findings could be due to the lower percentage of papillary structures and psammoma bodies observed in TC-PTC than in C-PTC (31, 32). Psammoma bodies correlate with microcalcifications, which are typical in C-PTC (33). Moreover, Ye et al. focused on developing a prediction model for TC-PTC, suggesting US characteristics (extra-thyroidal extension, aspect ratio ≥0.91, and maximum diameter ≥14.6 mm) for predicting TC-PTC (25). In addition, their analysis revealed no significant differences in vascular richness between TC-PTCs and C-PTCs under the Adler classification criteria. In the present study, blood flow distribution in TC-PTC was similar to that of C-PTC, typically showing peripheral or absent flow. In contrast, Hekimsoy et al. reported no significant difference between the US appearance of TC-PTC and C-PTC (13), probably related to a relatively small sample size.

HN-PTC, first adopted as an aggressive subtype in the 2017 WHO classification (34), is considered a moderately differentiated subtype with a high propensity for extrathyroidal extension, lymph node metastasis, and frequent carcinoma recurrence (35, 36). In the present study, patients with HN-PTC were the youngest among all PTC subtypes included, consistent with previous studies (26) and further supported by epidemiological data indicating a younger onset age for thyroid cancer (37). The US images in the HN-PTC group more frequently presented a wider-than-tall shape and the absence of extrathyroidal extension, similar to Ito et al., who reported that the patients with HN-PTC did not always present aggressive clinicopathological features at the initial surgery (38). Regardless of the stage at initial surgery, it is crucial for patients pathologically diagnosed with HN-PTC to undergo close and regular observation with blood examination and imaging studies after surgery. Notably, HN-PTC was characterized by internal vascularity, which significantly differs from C-PTC and TC-PTC. As Yang et al. reported (39), intranodular hypervascularity in malignant thyroid cancer may be related to the larger maximum size of the nodules compared to other subtypes. Additionally, Chen et al. introduced a novel pattern-based microvascular classification (PBMC) and subsequently constructed a multimodality US model that demonstrated significant diagnostic efficacy and clinical utility (40). These findings indicate the potential value of vascular imaging in diagnosing and differentiating thyroid nodules.

This study has some limitations. First, the retrospective study design may have culminated in selection bias; however, given the rarity of certain subtypes, prospective analysis was not feasible. Second, all study patients underwent thyroid surgery. Although this factor was necessary for correlating with the histopathological findings as a reference standard, sampling bias may have occurred. Third, specific subtypes (CC-, S-, and DS-) were excluded from further analysis due to the small sample size. These factors inevitably limit the generalizability of our results, as the representativeness of the cohort and the external validity of our conclusions may be constrained. Therefore, future multicenter prospective studies or pooled data analyses are warranted to ensure more balanced inclusion of rare subtypes and strengthen the robustness of conclusions.

## 5 Conclusions

In conclusion, US features of certain subtypes can help in the differential diagnosis of PTCs. Despite the overlap in several points of US manifestations, significant differences or some tendencies toward distinction were presented between C-PTC and aggressive PTC subtypes. In the era of personalized medicine and AS, acquaintance with the US characteristics of subtypes is of great importance for individualized treatment. Our results provide baseline information and highlight the need for future research in this field.

## Data Availability

The raw data supporting the conclusions of this article will be made available by the authors, without undue reservation.
