# Supplementary material for: Multimodal ultrasound features for distinguishing classic and aggressive subtypes of papillary thyroid carcinoma
Source: Front Endocrinol (Lausanne). 2025 Oct 1;16:1674109. doi: 10.3389/fendo.2025.1674109 (PMC12521462; doi:10.3389/fendo.2025.1674109)
Supplement: Supplementary file 1 [file DataSheet1.docx]

**Figure. S1. Flowchart of the study sample selection process.**

**
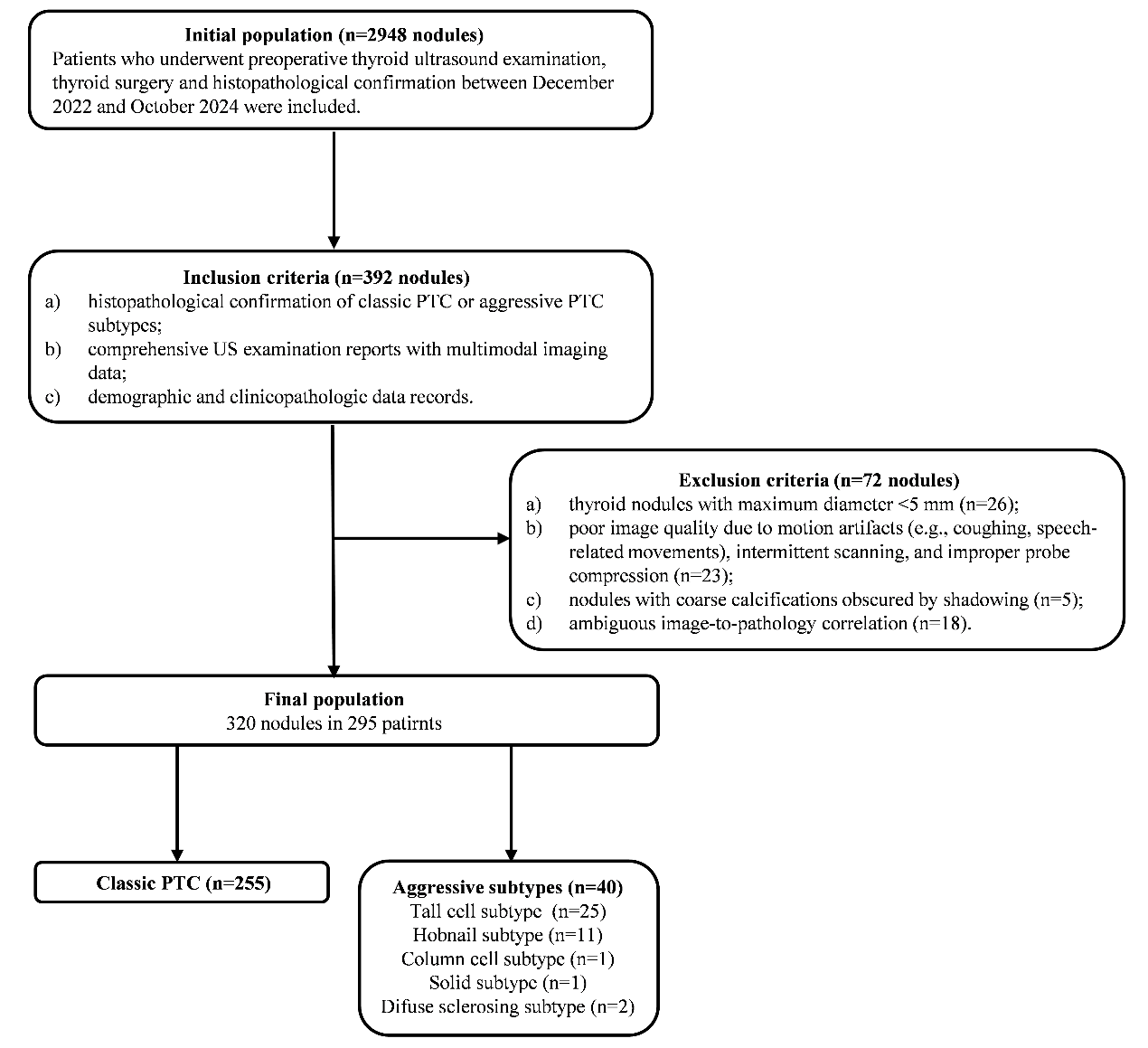
**

**Table S1. Standardized reporting of US features.**

| **Ultrasound features** | **Definition** |
| --- | --- |
| Composition |  |
| Solid | **·** No obvious anechoic cystic portions |
| Mixed solid and cystic | **·** Composed of solid tissue and a fluid component |
| Echogenicity |  |
| Hyperechoic or isoechoic | **·** Increased or similar echogenicity relative to the surrounding normal thyroid parenchyma |
| Mildly hypoechoic | **·** Decreased echogenicity relative to the surrounding normal thyroid parenchyma, but still increased echogenicity relative to the anterior neck muscles |
| Markedly hypoechoic | **·** Echogenicity less than or equal to the anterior neck muscle |
| Margin |  |
| Smooth | **·** Obviously discernible smooth edge |
| Ill-defined | **·** Poorly demarcated border of the nodule that cannot be differentiated from the adjacent thyroid tissue |
| Irregular | **·** Micro-lobulation or spiculation |
| Direction of growth |  |
| Wider-than-tall | **·** The anteroposterior diameter of a nodule is equal to or smaller than its transverse diameter in the transverse plane |
| Taller-than-wide | **·** The anteroposterior diameter of a nodule is greater than its transverse diameter in the transverse plane |
| Calcifications |  |
| None | **·** Absence of calcifications within the nodule |
| Macrocalcifications | **·** Large hyperechoic foci measuring >1 mm with posterior acoustic shadowing, in the absence of microcalcifications |
| Microcalcifications | **·** Punctate hyperechoic foci measuring ≤1 mm within the solid components of a nodule, in the absence of macrocalcifications |
| Mixed calcifications | **·** The coexistence of punctate and coarse hyperechoic foci within the same nodule |
| Extrathyroidal extension | **·** The invasion of the thyroid capsule and/or adjacent structures by direct extension from the thyroid nodule. |

**Table S2. C-TIRADS malignancy risk stratification of thyroid nodules.**

| **Category** | **US features** | **Points** | **Likelihood of malignancy** |
| --- | --- | --- | --- |
| C-TIRADS 1 | No nodule | – | 0% |
| C-TIRADS 2 | Benign | –1 point | 0% |
| C-TIRADS 3 | Probably benign | 0 point | <2% |
| C-TIRADS 4A | Low suspicion for malignancy | 1 point | 2%–10% |
| C-TIRADS 4B | Moderate suspicion for malignancy | 2 points | 10%–50% |
| C-TIRADS 4C | High suspicion for malignancy | 3–4 points | 50%–90% |
| C-TIRADS 5 | Highly suggestive of malignancy | 5 points | >90% |
| C-TIRADS 6 | Biopsy-proved malignancy | – | 100% |

**Table S3: Summary of multimodal ultrasonographic features across PTC subtypes.**

|  | **Composition** | **Echogenicity** | **Margin** | **Direction of growth** | **Calcifications** | **Extrathyroidal extension** | **CDFI** | **SWE (kPa)** |
| --- | --- | --- | --- | --- | --- | --- | --- | --- |
| Classic PTC | Solid (98.9%) | Mildly hypoechoic (81.4%) | Smooth (41.9%) | Taller-than-wide (53.4%) | None (54.1%) | Absent (75.3%) | I  (40.9%) | Emax 63.3 ± 37.3 Emean 44.1 ± 28.8 Emin 27.3 ± 20.8 |
| Tall cell subtype | Solid (100%) | Mildly hypoechoic (69.2%) | Irregular (53.8%) | Taller-than-wide (84.6%) | None (73.1%) | Present (46.2%) | II (38.5%) | Emax 69.6 ± 32.6 Emean 49.5 ± 26.5 Emin 31.0 ± 21.2 |
| Hobnail subtype | Solid (90.9%) | Mildly hypoechoic (72.7%) | Irregular (45.4%) | Wider-than-tall (72.7%) | Microcalcifications (63.6%) | Absent  (100%) | III (45.5%) | Emax 86.3 ± 60.5 Emean 60.1 ± 45.3 Emin 30.7 ± 16.6 |

PTC, papillary thyroid carcinoma; CDFI, color Doppler flow imaging; The vascularity of the nodules was categorized according to the CHAMMAS classification system: I, no signal blood flow; II, exclusively perinodular vascularity; III, perinodular blood flow ≥ central blood flow; IV, marked central blood flow and less marked perinodular blood flow; and V, exclusively central vascularity.

**Table S4. Pairwise comparisons among PTC subtypes for the direction of growth.**

| **Subtypes of PTC** | **C-PTC** | **TC-PTC** | **HN-PTC** |
| --- | --- | --- | --- |
| C-PTC | – | **0.005** | 0.372 |
| TC-PTC | **0.005** | – | **0.004** |
| HN-PTC | 0.372 | **0.004** | – |

PTC, papillary thyroid carcinoma; C-PTC, classic papillary thyroid carcinoma; TC-PTC, tall cell papillary thyroid carcinoma; HN-PTC, hobnail papillary thyroid carcinoma.

**Table S5 Pairwise comparisons among PTC subtypes for calcifications.**

| **Subtypes of PTC** | **C-PTC** | **TC-PTC** | **HN-PTC** |
| --- | --- | --- | --- |
| C-PTC | – | 0.372 | 0.318 |
| TC-PTC | 0.372 | – | **0.026** |
| HN-PTC | 0.318 | **0.026** | – |

PTC, papillary thyroid carcinoma; C-PTC, classic papillary thyroid carcinoma; TC-PTC, tall cell papillary thyroid carcinoma; HN-PTC, hobnail papillary thyroid carcinoma.

**Table S6 Pairwise comparisons among PTC subtypes for CDFI.**

| **Subtypes of PTC** | **C-PTC** | **TC-PTC** | **HN-PTC** |
| --- | --- | --- | --- |
| C-PTC | – | 1.00 | **0.007** |
| TC-PTC | 1.00 | – | **0.014** |
| HN-PTC | **0.007** | **0.014** | – |

CDFI, color Doppler flow imaging; PTC, papillary thyroid carcinoma; C-PTC, classic papillary thyroid carcinoma; TC-PTC, tall cell papillary thyroid carcinoma; HN-PTC, hobnail papillary thyroid carcinoma.

**Table S7 Pairwise comparisons among PTC subtypes for extrathyroidal extension.**

| **Subtypes of PTC** | **C-PTC** | **TC-PTC** | **HN-PTC** |
| --- | --- | --- | --- |
| C-PTC | – | 0.101 | 0.216 |
| TC-PTC | 0.101 | – | **0.020** |
| HN-PTC | 0.216 | **0.020** | – |

PTC, papillary thyroid carcinoma; C-PTC, classic papillary thyroid carcinoma; TC-PTC, tall cell papillary thyroid carcinoma; HN-PTC, hobnail papillary thyroid carcinoma.

**Table S8 Effect size for the association between ultrasonographic features and PTC subtypes.**

| **Subtype US features** | **p value** | **Cramer’s V** | **95% CI** |
| --- | --- | --- | --- |
| Direction of growth | **0.002** | 0.20 | 0.08 – 0.31 |
| Calcifications | NA | – | – |
| Extrathyroidal extension | **0.008** | 0.17 | 0.05 – 0.28 |
| CDFI | **0.017** | 0.17 | 0.03 – 0.22 |

Cramer’s V was calculated for the effect size, indicating the strength of the association, which was categorized as follows: weak (0.1-0.2), moderate (0.2-0.4), or strong (0.4 or greater). NA indicates that the effect size could not be reliably estimated due to sparse data distribution.

**Table S9. Interobserver agreement for US features.**

| **US feature** | **Kappa** | **p value** | **Agreement Level** |
| --- | --- | --- | --- |
| Composition | 1.00 (1.00-1.00) | <0.001 | Almost perfect |
| Echogenicity | 0.66 (0.42-0.87) | <0.001 | Substantial |
| Margin | 0.63 (0.46-0.79) | <0.001 | Substantial |
| Direction of growth | 0.85 (0.71-0.97) | <0.001 | Almost perfect |
| Calcifications | 0.63 (0.48-0.79) | <0.001 | Substantial |
| Extrathyroidal extension | 0.78 (0.58-0.93) | <0.001 | Substantial |
| CDFI | 0.69 (0.53-0.83) | <0.001 | Substantial |

Data in brackets are 95% confidence intervals. Agreement level: Kappa ≤ 0.8, Moderate: 0.4 < Kappa ≤ 0.6, Fair: 0.2 < Kappa ≤ 0.4, Slight: 0 < Kappa ≤ 0.2, Poor: Kappa ≤ 0. ICC, intraclass correlation coefficient; US, ultrasound; CDFI, color Doppler flow imaging.
